# When science comes before progress

**DOI:** 10.1038/s41531-019-0082-8

**Published:** 2019-06-03

**Authors:** Benjamin Stecher

**Affiliations:** Unaffiliated, Toronto, Canada

**Keywords:** Conferences and meetings, Neuroscience

For 5 days in late March 2019, nearly 4000 researchers made their way to Lisbon, Portugal for the 14th International Conference on Alzheimer’s & Parkinson’s Diseases. I was diagnosed with Parkinson’s disease 6 years ago at age 29, and have spent the past 3 years traveling the world asking questions of every expert possible to get more information about what is going on inside my own head. This journey led to my being invited to speak at this conference and to see firsthand what these scientific conferences are like. What follows is my perception of the conference, as an invited Parkinson’s disease patient advocate.

This year’s meeting came on the back of some somber news. The week prior, it was announced that another phase III clinical trial drug designed to target the build-up of amyloid beta, the protein thought to be the cause of Alzheimer’s disease, had failed.^1^ Not only did it fail, it failed a futility analysis.^2^ This indicates that there was something catastrophically wrong with either the selected target, the ability of the drug to hit that target, or the design of the trial.

Though devastating, the timing of this announcement presented a perfect opportunity for those in attendance to start asking some difficult questions, and to critically examine the reasons why the field has nothing but failures to show in its attempts to modify the progression of these diseases. However, what we got instead was just apologetic overtures at the outset of a couple talks from representatives of the company that ran the trial before a return to the regularly scheduled programming.

At the root of the problem is the reason why so many traveled so far to come to this conference. In short, they came to learn what their colleagues and competitors are working on, to promote their science, and to find collaborators or funders that could help push forward whichever narrow domain of study they happen to be pursuing. In my view, that is science today, it is how the game is played, and how careers are made.

There is nothing inherently wrong with that, unless one follows the source of the funding. A great deal of research in this field appears to be paid for by public institutions, patient organizations, and charitable foundations. The researchers that receive funding from these bodies should be directly accountable to these groups, and work towards what is in the best interests of the patients and societies they represent. The difficulty there is that a great deal of research is too esoteric and complex for almost anyone but fellow experts to understand. Though almost everyone in the field has the best of intentions, science is still a human endeavor, and it is not immune to our shortcomings or our self-interested nature. However, I believe the stakes in biomedical research are too high to allow for this status quo to persist. Doing so perpetuates a culture that caters to researchers’ careers rather than the welfare of the society and patients that fund it, and renders advancing treatment paradigms a by-product rather than the goal of medical science.

This culture of science for scientists was evident just by looking at the list of invitees to the meeting.^3^ There was only one person living with either Alzheimer’s or Parkinson’s invited to speak, me, and that invitation was from an industry sponsor, not the academic organizers of the event. Not only does keeping patients at a distance disassociate researchers from the realities of how these diseases manifest in humans, but it deprives the scientific community of the unique perspectives that only those living with the disease can provide. Patients are the only ones capable of tapping into the knowledge to be gained from the lived experience. Additionally, they come from rich and varied backgrounds, which would add more sorely needed diversity of opinion to the field.

I believe implementing these changes will be critical steps towards accelerating progress…

Another puzzling occurrence was that many of the talks either began or ended with the speaker thanking patients for giving samples or agreeing to donate their brains. I was left to wonder who they were addressing, since there were no other patients in attendance to my knowledge. If researchers truly wanted to thank the patients, it would not be with empty words of acknowledgment, but by putting the interests of those who had agreed to donate parts of themselves ahead of their scientific careers.

To quote Prof. Hilal Lashuel from an email exchange I had with him after attending the conference, “Any practices that we are not able defend or justify in front of taxpayers and patients is something we should not do in our lab. It is that simple and the least we should do to fulfill our contract with society and to express our appreciation to them for their trust and continued support.”

These problems appear to be even further exacerbated by the clandestine atmosphere at these conferences, and how data relevant to the community is presented. Many sessions opened with a stern reminder not to take pictures. Why? Because researchers are worried about being scooped, they don’t want their hard work stolen by a competitor before they have had time to secure the publication needed for them to get proper credit. Much of the fault for this practice lies in our institutions because almost all research centers use publication history as a measure of performance. This puts researchers into a position where many may feel forced to put the needs of their careers over the needs of the people they are trying to help.

So, how can we start to address these systemic problems and nudge the culture of medical science towards one more in line with the needs of patients and society? As a first step I propose that all medical science conferences and research centers move to adopt the following policies:

1. Adhere to the spirit and principles of open science.
2. Recognize and include informed patients and caregivers as a valued resource.

I believe implementing these changes will be critical steps towards accelerating progress in the field and bring us closer to a day when at a future Alzheimer’s or Parkinson’s disease meeting, instead of mourning yet another failure, we will finally have reason to celebrate some success.
